# The nociceptin/orphanin FQ receptor partial agonist sunobinop promotes non-REM sleep in rodents and patients with insomnia

**DOI:** 10.1172/JCI171172

**Published:** 2024-01-02

**Authors:** Garth T. Whiteside, Donald J. Kyle, Ram P. Kapil, Alessandra Cipriano, Ellie He, Mingyan Zhou, Manjunath S. Shet, Michele Hummel, Terri Knappenberger, Kazuya Fukumura, Yoshiyuki Matsuo, Masahiro Uehira, Shuichi Hiroyama, Nozomi Takai, Sandra K. Willsie, Stephen C. Harris

**Affiliations:** 1 Imbrium Therapeutics, Stamford, Connecticut, USA.; 2 Purdue Pharma LP, Stamford, Connecticut, USA.; 3 Shionogi & Co. Ltd., Osaka, Japan.; 4 Pharmaceutical Research Associates, Raleigh, North Carolina, USA.

**Keywords:** Neuroscience, Therapeutics, Pharmacology

## Abstract

The potent and partial agonist sunobinop activates the nociceptin/orphanin-FQ peptide receptor and promotes non-REM sleep in rodents and patients with insomnia.

**To the Editor:** Insomnia is a common disorder and public health burden. Frequently prescribed hypnotics enhance γ-aminobutyric acid or block orexin signaling. With few pharmacological modalities and limitations associated with traditional targets, there is high interest in new mechanisms involved in sleep/wake regulation. The utility of the nociceptin/orphanin FQ receptor (NOP) system as a treatment for insomnia in humans has not been described. We utilized a potent and selective partial agonist that originated from our laboratories, sunobinop (1), to study the role of NOP in sleep/wake in rats and human patients; this is the first manuscript, to our knowledge, that describes the pharmacology of sunobinop.

We characterized the in vitro, ex vivo, and in vivo pharmacology and pharmacokinetics of sunobinop in cells stably overexpressing human NOP, rat brain sections, and rats. We utilized EEG to characterize sleep/wake and behavioral assays to characterize learning and memory, reward, respiration, and intestinal transit. We determined safety, tolerability, and pharmacokinetics in a cohort of 18 healthy male human subjects and assessed sleep using polysomnography in 22 patients with insomnia disorder (for detailed description of procedures, see Supplemental Methods; supplemental material available online with this article; https://doi.org/10.1172/JCI171172DS1).

Sunobinop is a potent partial agonist at human NOP receptors with high affinity (K_i_ of 3.3 ± 0.4 nm; EC_50_ of 4.03 ± 0.86 nm, E_max_ of 47.8% ± 1.31%) (Supplemental Figure 1A). Sunobinop competitively inhibited [^3^H]-NOP-1A binding in rat-brain sections (IC_50_ of 7.7 nM) and achieved an in vivo NOP receptor occupancy in the hypothalamus of 74.7% ± 11.3% at 30 mg/kg (Supplemental Figure 1, B and C). Importantly, sunobinop does not activate human μ and κ receptors and is a low-affinity, weak partial agonist at human δ receptors (Supplemental Figure 1A). After oral administration to rats (0.3–30 mg/kg), plasma concentration reached a maximum at 2.50–4.50 h with C_max_ and AUC_inf_ values that increased dose proportionally and bioavailability that ranged from 31.2% to 42.1%. (Figure 1A). Administration of 30 and 300 mg/kg of sunobinop induced obvious EEG changes in rats; wakefulness was significantly decreased (*P* < 0.01). Conversely, non–rapid eye movement (non-REM) sleep was significantly increased (*P* < 0.01). A non–dose-dependent effect on REM sleep was noted (significant at 30 but not 300 mg/kg) (Figure 1B). To confirm these effects were mediated via activation of NOP, 300 mg/kg of sunobinop was administered to NOP-KO rats and the EEG changes observed in WT were near abolished (Figure 1B and Supplemental Figure 2B). Zolpidem induced a short-lived significant increase in non-REM sleep, indicating an intact functional GABAergic system in the knockouts (Supplemental Figure 2C). No statistically significant treatment-related changes were noted in rat assays of learning and memory, reward, respiration, and intestinal transit (Figure 1, C and D, and Supplemental Figure 1, D and E) at doses substantially above those required for a therapeutic effect.

In healthy male human subjects, sunobinop exhibited rapid absorption after oral administration across a wide dose range (3–30 mg) and a half-life of 2.1–3.2 h, suggesting suitability for once-daily dosing at nighttime with low concentrations present the following morning. Beyond 10 mg, systemic exposure increased less than dose proportionally (Figure 1E), with a lower percentage of unchanged drug recovered in urine (70% at 10 mg; 28% at 30 mg; 89% at 3 mg) and no detectable levels of metabolites identified in plasma or urine, suggesting dose-limiting absorption and a predominantly renal route of excretion of absorbed drug.

In patients with insomnia disorder, sleep efficiency (SE), the primary end point, was significantly higher after dosing with sunobinop (10 mg) than after placebo (91.4% versus 79.8%, respectively). The drug-effect difference between sunobinop and placebo was 11.8% (*P* < 0.0001). Sunobinop also produced a reduction in latency to persistent sleep (LPS) (*P* = 0.0136), less wake after sleep onset (WASO) (*P* < 0.0003), and fewer nighttime awakenings (*P* < 0.0001). Sleep-stage analysis revealed little-to-no change in the placebo-treated subjects, while sunobinop-treated subjects had less stage N1 sleep, more stage N2 sleep, a reduced REM period, and no significant change in stage N3 sleep or REM latency (Figure 1, F and H). Sunobinop also increased perceived sleep quality (*P* = 0.002) (Figure 1G).

Sunobinop was generally well tolerated in healthy subjects and patients (Supplemental Table 1, A and B). There were no deaths, serious adverse events (SAEs), or discontinuations due to adverse events (AEs). The most commonly reported treatment-emergent events were fatigue/somnolence (following 10 and 30 mg, 1 of 4 subjects experienced somnolence sufficient to interfere with activities of daily living), euphoria, and dizziness in healthy subjects and somnolence/sedation in patients. Sunobinop did not produce clinically relevant changes in hematology, chemistry, and urinalysis results, and no meaningful changes from baseline were observed in ECGs and SpO_2_.

We conclude that a 10 mg oral dose of sunobinop has a large positive effect on sleep/wake function in subjects with insomnia, thus providing a more consolidated, quality sleep. However, observations of next-day residual effects, as supported by the frequency of reported somnolence AEs, indicate that dose-ranging studies are needed to define the optimal effective dose (for additional details on findings, see Supplemental Results). Our translational research demonstrates that activation of NOP represents an additional mechanism and an attractive treatment approach for insomnia disorder in humans. Sunobinop’s profile is suitable for continued clinical development, and several additional clinical studies have been initiated, including a phase 2 trial in patients suffering from insomnia during recovery from alcohol use disorder (ClinicalTrials.gov NCT04035200) (2).

## Supplementary Material

Supplemental data

Supporting data values

## Figures and Tables

**Figure 1:**
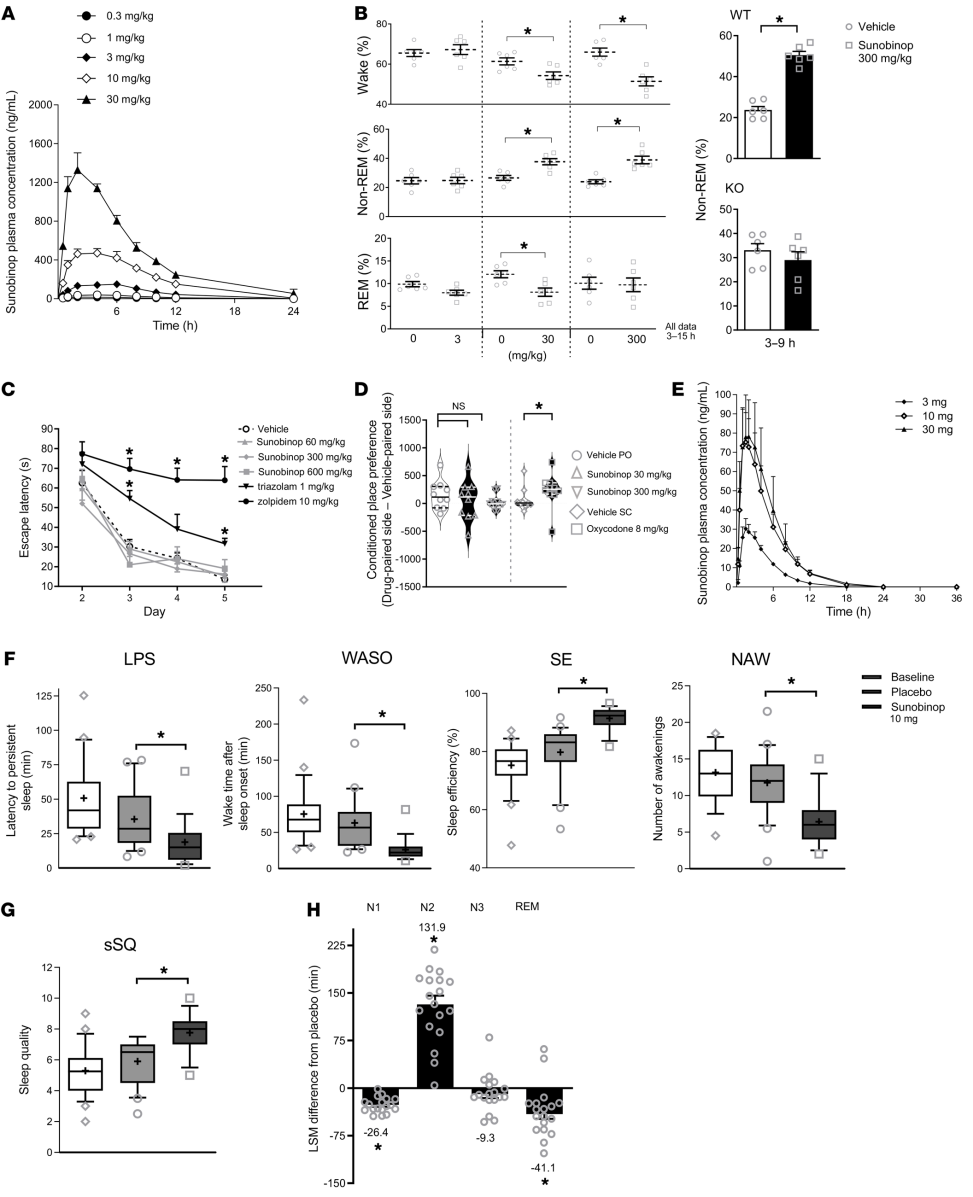
Sunobinop pharmacokinetics in rat and human, lack of reward and lack of effect on learning and memory in rats, and effects on sleep parameters in rats and human patients. (**A**) Pharmacokinetic profile in rats after oral dosing. (**B**) Effect on sleep stages as measured by EEG in WT and NOP-KO rats. (**C** and **D**) Effect on learning and memory and reward in rats as measured by Morris water maze and conditioned place preference. (**E**) Pharmacokinetic profile in healthy male human subjects after oral dosing. (**F**–**H**) Polysomnography results in patients with insomnia disorder: (**F**) sleep parameters, subjective sleep quality (sSQ) (**G**), and sleep architecture (**H**). NAW, number of awakenings. Data are represented as means ± SEM (**A**–**C** and **H**), means ± SD (**E**), and median ± interquartile range (**D**, **F**, and **G**), with dotted lines (**D**) and error bars (**F** and **G**) showing 10th to 90th percentiles, outliers shown, and plus signs indicating the mean (**F** and **G**). Data points in **H** represent raw data. *P ≤ 0.05, ANOVA (**D**); *t* test (all others).

## Version 1 10/26/2023

In-Press Preview

## Version 2 01/02/2024

Electronic publication
