# Supplementary material for: The nociceptin/orphanin FQ receptor partial agonist sunobinop promotes non-REM sleep in rodents and patients with insomnia
Source: J Clin Invest. 2024 Jan 2;134(1):e171172. doi: 10.1172/JCI171172 (PMC10760950; doi:10.1172/JCI171172)
Supplement: Supplemental data [file jci-134-171172-s272.pdf]

Supplemental Material for:

The nociceptin/orphanin-FQ receptor partial agonist sunobinop promotes non-REM sleep in rodents and patients with insomnia

Garth T. Whiteside[1*], Donald J. Kyle[2], Ram Kapil[1], Alessandra Cipriano[1], Ellie He[1], Mingyan Zhou[1], Manjunath Shet[1], Michele Hummel[2], Terri Knappenberger[2], Kazuya Fukumura[3], Yoshiyuki Matsuo[3], Masahiro Uehira[3], Shuichi Hiroyama[3], Nozomi Takai[3], Sandra Willsie[4†], Stephen Harris[1]

SUPPLEMENTAL METHODS

Animals

For in vivo assays we used male outbred male Sprague Dawley rats (Charles River). We also used male wild type and NOP knockout rats out on a Wistar Hannover background (Transposagen Biopharmaceuticals). Rats were 6-8 weeks old at time of dosing except for NOP knockout rats and controls which were 12 weeks old. Rats were group housed and kept on a 12 h:12h light:dark schedule at an ambient temperature (20-24°C). Rats were randomly assigned to experimental groups and behavioral tests were conducted by researchers blinded to treatment group.

Solutions and Materials

We used sunobinop (Shionogi, Osaka; Purdue, Cranbury NJ; Albany Molecular Research, NY) suspended in 0.5% methyl cellulose for oral dosing to rats and humans. For tissue binding studies [^{3}H]-NOP-1A (Quotient Bioresearch Ltd.) with a specific radioactivity of 2890 GBq/mmol was diluted in 50 mM Tris-HCl buffer containing 5 mM $MgCl_2$, 1 mM EDTA, and 0.1% BSA (pH 7.4) to prepare a solution with a final concentration of 8 nM which was subsequently serially diluted; sunobinop was dissolved in dimethyl sulfoxide (DMSO) to prepare a 1 mM solution which was subsequently serially diluted. Heparin sodium

(1000 U/mL, JP standard) purchased from Ajinomoto Co., Inc. was used. All other materials were purchased from Sigma Aldrich (MO).

In vitro receptor pharmacology

Sunobinop was tested for binding affinity and functional response in NOP, MOP, KOP and DOP assays using recombinant HEK-293 cell lines separately expressing human NOP, MOP, KOP and DOP receptors as previously described (1). [^{3}H]-OFQ/N, [^{3}H]-DAMGO, [^{3}H]-U69,593 and naloxone were used for NOP, MOP, KOP and DOP receptors, respectively, in the competition binding assays. Functional response in the GTPgS assays for NOP, MOP, KOP and DOP were normalized to 10 µM N/OFQ, 10 µM DAMGO 10 µM U69,593 and 10 µM met-enkephalin, respectively, where the response of these reference agonists at 10 µM = 100% E_{MAX}. The data from competition binding and functional response experiments are presented as mean values ± S.E.M., based on at least three repeat tests per molecule. Data were analyzed using Microsoft Excel and the curve fitting functions in GraphPad PRISM™, v.7.0.

Ex vivo brain binding

To confirm that sunobinop bound to rat NOP receptors an ex vivo competition binding study was conducted using the selective radiolabeled NOP agonist [^{3}H]-NOP-1A (2, 3). Rat brain sections were preincubated for 15 minutes at 25°C in assay buffer and then incubated in a solution of radiolabeled ligand, in the absence (total binding) or the presence of competing compound (unlabeled-NOP-1A for nonspecific binding, or sunobinop) for 120 minutes at 25°C in duplicate. Sections were immersed twice in ice-cold wash buffer (50 mM Tris-HCl containing 5 mM $MgCl_2$ and 1 mM EDTA, pH 7.4) for 10 minutes, rinsed in ice-cold distilled water, and dried under a cool stream of air. Binding determination was performed in duplicate. Sections were exposed to an imaging plate for ^{3}H-labeled compound (BAS-TR2040, GE Healthcare) together with [^{3}H]microscale (ART 0123, American Radiolabeled Chemicals, Inc.) at room temperature for 8-9 days. After exposure, the imaging plate was removed and scanned with a

bio-imaging analyzer (Typhoon FLA 9500, GE Healthcare). Dissociation constant (K_d) and maximal number of binding site (B_{max}) of [^{3}H]-NOP-1A were calculated from saturation binding data. Fifty-percent inhibitory concentration (IC_{50}) of sunobinop was calculated from competition binding data using GraphPad Prism version 5.

In vivo receptor occupancy

To confirm that sunobinop occupied rat NOP receptors in vivo a receptor occupancy study was conducted. Sunobinop was administered p.o. at 3, 30 and 300 mg/kg (n=6/group); 3 h post-dosing, [^{3}H]-NOP-1A (506kBq/0.2ml/rat) was administered intravenously and 1 h later rats were sacrificed by decapitation under isoflurane anesthesia the brain removed and the hypothalamus and striatum dissected (blood was also collected at the same time). Brain samples were solubilized and mixed with 10 mL of pico-fluor. The radioactivity of each sample was measured with a liquid scintillation analyzer (TriCarb 3100TR, PerkinElmer, Inc.). Plasma concentration of radioactivity was expressed as percent injected radioactivity per gram (%dose/g). The radioactivity concentration expressed as percent injected radioactivity per gram (%dose/g) was calculated in each brain region. The striatum was used as the reference region for estimation of the free and non-specific binding fraction (4). Specific binding was calculated for individuals by subtracting the concentration of radioactivity in the striatum from the concentration of radioactivity the hypothalamus. The receptor occupancy was calculated as follows: Receptor occupancy (%) = 100 × [1 – (specific binding in test substance-treated rat)/(mean specific binding in vehicle-treated rats)]. The mean value of specific binding in vehicle-treated rats and the individual value of specific binding in test substance-treated rat were used for the receptor occupancy calculation.

Pharmacokinetics in rats

To confirm sunobinop displayed pharmacokinetics suitable for further in vivo testing rats were dosed (0.3-30 mg/kg, p.o. n=4 per group) and blood (approximately 0.2 mL) was collected from a jugular cannula using a heparinized 1-mL syringe attached with a 23G needle (both from Terumo Corporation). The blood was immediately centrifuged at 14000 rpm for 1 min at 4°C using a centrifuge (TOMY MX-100) to obtain plasma. Approximately 50 µL of the plasma was transferred into a cluster tube and stored in a freezer at ~-20°C. The supernatants, obtained by protein precipitation of rat plasma (30 µL) with methanol, were analyzed by LC/MS/MS (API5000 system) with *m/z* transition of 436.2-246.5 and limit of quantification of 0.5 ng/ml. The pharmacokinetic parameters were calculated using WinNonlin (version 5.0.1, Pharsight Corp.) based on a non-compartment model with uniform weighting.

Electroencephalography

To study the effects of sunobinop (3-300 mg/kg, p.o., n=6/group) on sleep/wake in rats, electrodes were chronically implanted into the cortex (via stereotaxic surgery) and dorsal neck muscle. Electroencephalogram (EEG) and electromyogram (EMG) were recorded via telemetry with 5 EEG levels analyzed visually. Under isoflurane anesthesia, the radio telemetry transmitter (TLM10M3-F50-EEE, Data Sciences International) was implanted into the abdominal cavity and the lead lines were let out to the dorsal neck through a subcutaneous tunnel. The screw electrodes were placed on the dura matter over the right and left parietal cortex and occipital cortex: the coordinates used were 1.8 mm posterior to bregma, 3.5 mm lateral from the midline suture and 5.2 mm posterior to bregma, 2.0 mm lateral from the midline suture, respectively. A pair of stainless wires for electromyogram recording were inserted into the dorsal neck muscle. Each electrode was soldered to a lead line of the telemetry transmitter, which was fixed on the skull with quick self-curing acrylic resin (ADFA, Shofu Inc.) and animals allowed a minimum of 5 days recovery prior to testing. EEG activities were recorded for ~15 h post-dosing (8 h post-dosing with zolpidem) using EEG recording software (Dataquest A.R.T., Data Sciences International). The sampling frequency and filtering mode for EEG and EMG were set at 250 and 0.1-30 Hz (band pass) and 1000 Hz

and 5 Hz (low cutoff), respectively. Sleep stages were divided into 5 stages; wakefulness, rest, non-REM (split into slow wave light sleep and slow wave deep sleep) and REM sleep. Each stage was adjudicated every 20 seconds by visual inspection according to “A manual of standardized terminology, techniques and scoring system for sleep stages of human subjects” (5) by a single experienced researcher.

Whole body plethysmography

To determine if activation of NOP produced effects on respiratory parameters sunobinop (60-600 mg/kg, p.o., n=8/group) was administered and effects on respiratory rate and ventilation volume assessed in unanesthetized rats using unrestrained whole-body plethysmograph (WBP) system (Bio System-XA, Buxco Electronics, Inc.). Respiratory signals were recorded every 5 seconds.

Charcoal meal assay of gastrointestinal transit

To determine if activation of NOP produced effects on small intestinal motility sunobinop (10-600 mg/kg, p.o., n=8/group) was administered. Morphine hydrochloride (5 mg/kg, s.c.) served as a positive control. 0.5 mL of 5 w/v% activated charcoal suspended in a 10 w/v% arabic gum solution was orally administered 4 h after sunobinop and 30 mins after morphine (based on respective pharmacokinetics). Animals were euthanized 20 min after oral administration of the activated charcoal suspension, and immediately subjected to celiotomy. After marking the forefront of the transported activated charcoal with a clip, the stomach and small intestine were isolated by cutting off the intestinal membrane. The length of the small intestine between the ileocecum and forefront of the transported activated charcoal, and the full length of the small intestine from the pylorus to ileocecum was measured. The transit rate was expressed as a percentage of total length.

Morris Water Maze

To assess the influence of activation of NOP on spatial learning and memory, sunobinop (60- 600 mg/kg, p.o., n=7-10/group) was tested using the Morris water maze test in rats. Triazolam (1 mg/kg, p.o.) and zolpidem (10 mg/kg, p.o.) served as positive controls. All compounds were administered once a day for 5 days. Administration was performed on the days of Morris water maze test (including the day of habituation to water) prior to measurement. The Morris water maze test was performed 2 h (sunobinop) or 30 minutes (Positive controls) after dosing (based on respective time to reach C_{max}). Rats were acclimated to the test environment (round pool of 150 cm in diameter, 45 cm in height and containing water at depth of 30 cm with a colorless and transparent platform of 12 cm in diameter set ~1 cm below the water surface) with free swimming for 90 seconds without an escape platform on the first administration day (Day 1). Measurements were performed using a video-tracking system (Smart, Panlab) with 3 trials per day at an interval of 30 minutes from Day 2 to 5 with escape latency being the evaluation parameter. The starting point was changed at each trial, but the platform position was kept constant in all sessions. The maximum swimming time permitted per trial was 90 seconds, after which all rats that failed to reach the platform were placed on the platform for 30 seconds.

Conditioned Place Preference

To determine if activation of NOP produced rewarding effects sunobinop (30-300 mg/kg, p.o., n=9-10/group) was tested in a conditioned place preference (CPP) assay as compared to vehicle treated rats. Oxycodone (8 mg/kg, s.c.) served as the positive control. In the CPP model, an unbiased paradigm was used in which rats were randomly conditioned to test compounds and vehicle compartments. An automated three compartment chamber (MedAssociates, Fairfax, VT) was used in the assessment such that animals received six compartment pairings (3 drug: 3 vehicle) on alternate days between the two chambers. Control animals received vehicle at each of the six conditioning sessions. Conditioning sessions lasted 30 minutes and commenced 30 minutes after vehicle or test compound administration. CPP was

determined a day following the last conditioning session and is represented as the difference between the time spent (in seconds) in the drug-paired and the vehicle-paired chambers on the test day.

Pharmacokinetics in humans

The pharmacokinetics, safety, and tolerability of sunobinop in humans were evaluated in a randomized, double-blind, placebo-controlled, escalating single oral dose administration in healthy male subjects. Subjects were nonsmoking healthy male subjects aged 18 to 45 years, inclusive, who exhibited no clinically relevant medical history or disease and were deemed suitable to take part in this clinical study by the investigator. Subjects with history of obstructive uropathy and/or renal disease/dysfunction or with any cardiovascular disorder were excluded. Sunobinop was administered as an oral aqueous suspension to sequential dose cohorts (1, 10, and 30 mg) of 6 subjects each (4 receiving active treatment and 2 receiving placebo). Blood samples for determining sunobinop plasma concentrations were obtained from each subject at predose (within 30 minutes prior to dosing) and at 0.25, 0.5, 1, 1.5, 2, 3, 4, 6, 8, 10, 12, 18, 24, 36, 48, 60, 72, 84, and 96 h post dose. Plasma concentrations of sunobinop were quantified by a validated liquid chromatography tandem mass spectrometric (LC-MS/MS) method. Pharmacokinetic parameters were calculated using WinNonlin (version 5.0.1, Pharsight Corp.) based on a non-compartment model with uniform weighting.

Polysomnography

An impact on sleep wake function was established in a phase 1, randomized, single-center, double-blind, placebo-controlled, crossover proof of concept (POC) study that assessed the effects of sunobinop on sleep efficiency in subjects (n=22) with insomnia disorder. Subjects were non-smoking males or females 18 to 60 years of age inclusive with insomnia disorder. Subjects were healthy and free of clinically relevant abnormal findings as determined by medical history, physical examination, clinical hematologic and serum

chemistry laboratory values, vital signs and electrocardiogram with the exception of insomnia. Insomnia disorder as defined by the Diagnostic and Statistical Manual of Mental Disorders, Fifth Edition (DSM-5) was established based on medical history and assessments performed at screening visits and confirmed by polysomnography during the screening visit as a mean latency to persistent sleep less than or equal to 20 minutes, a mean wake after sleep onset of greater than or equal to 20 minutes and a total sleep time of less than 420 minutes. The study included 3 periods: pre-randomization (up to 28 days), treatment (10 days), and a phone follow-up safety evaluation. The 10-day treatment period included 2 dosing periods (dosing periods 1 and 2) that were approximately 5 days apart. Dosing periods 1 and 2 each consisted of a stay of 2 consecutive nights, during which subjects received the same study drug (either 10 mg sunobinop or placebo) on both evenings of that dosing period. Study drug was administered 30 minutes before the subject's median habitual bedtime (to the nearest quarter hr, as determined by a sleep diary) in each dosing period according to the study randomization schedule. Subjects were randomized to treatment sequence upon check-in on day 1 once continued eligibility was confirmed. Results from the day 1 and day 8 check-in laboratory assessments were reviewed by the principal investigator (or designee) before dosing the subject for that dosing period. Following the evening-time dosing with study drug, subjects underwent 8 h of continuous polysomnography (PSG) recording. Next-day residual effects were assessed starting at approximately 30 minutes after lights-on. All tests were administered in the clinical unit, starting 30 minutes after lights-on and every 90 minutes thereafter for approximately 16 h post-lights-out, following PSG recordings. The full analysis population was used for mean sleep efficiency, latency to persistent sleep, wake after sleep onset and total duration of N1, N2, N3 and rapid eye movement (REM) sleep stage. PSG measured sleep components were derived by taking the mean of measure for prerandomization as baseline. The 2 PSG nights in each dosing period (day 1 and day 2, day 8 and day 9) were averaged before comparison. Additionally sleep quality was recorded using a scale from 1 to 10, where 1 is poor and 10 is excellent.

Statistics

For electroencephalography statistical analyses were performed with paired Student's t-test compared to vehicle using SAS system (Version 9.4, SAS Institute Japan) with 0.05 level of significance; note we reported both unadjusted p-values and p-values following Bonferroni correction in time course experiments. For whole body plethysmography, gastrointestinal transit, grip strength and Morris water maze differences from the control group were evaluated at the two-tailed 0.05 level of significance using Dunnett's test (whole body plethysmograph) or Students t-test (all others). For conditioned place preference statistical analysis (one-way analysis of variance) with 0.05 level of significance was performed in GraphPad Prism version 5.

No formal sample size calculations were performed for the single oral dose administration pharmacokinetic study in humans. The sample size was based on medical and clinical judgement and considered adequate to achieve study objectives. Each cohort sample was 6 subjects (4 subjects randomized to active drug and 2 subjects randomized to placebo), which was a 2:1 randomization schema. Demographic, baseline, safety, pharmacokinetic and pharmacodynamic findings were summarized using descriptive statistics. Pharmacokinetic analyses were based on the full analysis population which included all randomized patients that received study drug and had at least one quantifiable pharmacokinetic sample.

In the cross over design polysomnography study, a sample size of 24 subjects was estimated to have 90% power to detect a difference in SE means of approximately 7%, assuming a standard deviation (SD) of differences of 10, using a 2-group t-test (crossover analysis of variance [ANOVA]) with a 0.05 two-sided significance level. In the literature (6), the SD of the differences between active and placebo is approximately 9. Since there was no prior information on the active dose of sunobinop for SE, an SD of 10 was used as a conservative estimate of dispersion. The full analysis population for the primary and

secondary efficacy outcomes was the group of subjects who were randomized and received 1 dose of the study drug. Analysis was based on assigned treatment. The statistical analysis to compare sunobinop with placebo was performed by using a mixed-model approach that included period, sequence, and treatment as fixed effects; subject within the sequence as a random effect; and the baseline measurement as a covariate. The postbaseline observed value was the response variable. The 2-sided significance level of 0.05 was used for comparison. The difference between treatments and its 95% confidence interval was also calculated. Statistical analysis was performed by using SAS®, Version 9.3. Demographic, baseline, safety, pharmacokinetic and pharmacodynamic findings were summarized using descriptive statistics.

The safety population for both the pharmacokinetic and polysomnography studies included all subjects that were randomized and received at least one dose of study medication. Subjects' AEs for the randomized safety populations were categorized into preferred terms and associated system organ class (SOC) using the Medical Dictionary for Regulatory Activities (MedDRA). Treatment-emergent AEs (TEAEs) were defined as AEs that started or increased in severity after the first dose of study drug. TEAEs were summarized by presenting the incidence of AEs for each treatment group by the MedDRA preferred term, nested within SOC.

Study Approval

All animal protocols were reviewed and approved by the Institutional Animal Care and Use Committees relevant to each study site and were conducted in accordance with the 2011 Guide for the Care and Use of Laboratory Animals. All clinical studies in humans were conducted in accordance with GCP under an FDA approved IND and were approved by the Midlands (protocol OAG1001) and New England (protocol OAG1003) Institutional Review Boards; subjects provided their written consent to participate in the study after having been informed of the nature and purpose of the study, participation/termination conditions, and risks and benefits of treatment.

Data Availability

Values for all data points shown in graphs and values behind the reported means are provided in the Supporting Data Values file.

SUPPLEMENTAL RESULTS

Sunobinop is a potent partial and selective agonist at human NOP receptors in vitro, potently binds to NOP in coronal sections of rat brain ex vivo and occupies the receptor in vivo

Sunobinop was characterized at human NOP, mu, kappa and delta receptors overexpressed in HEK-293 cells; sunobinop displayed a high affinity for human NOP receptors with a Ki of 3.3±0.4 nm and was determined to be a potent partial agonist with EC_{50} of 4.03±0.86 nm and Emax of 47.8±1.31%. Sunobinop was characterized to have low affinity at mu and kappa receptors (Ki 1630±76.5 nM and 2280±213 nm, respectively) without agonist activity (up to 10 µM) and a weak agonist at delta receptors (Ki 4763±509 nm, EC50 2205±396.6 nM, Emax 16.4±0.75%) (Supplemental Figure 1A).

[^{3}H]-NOP-1A bound to the rat brain cerebral cortical sections with a Kd of 9.2 nM and a Bmax of 66.0 fmol/mg tissue. Sunobinop competitively inhibited [^{3}H]-NOP-1A binding in rat cortical brain sections (Supplemental Figure 1B) with an IC_{50} of 7.7 nM. These data demonstrate that sunobinop competitively inhibits [^{3}H]-NOP-1A binding in rat brain tissue and confirms that it potently binds to NOP ex vivo. Sunobinop NOP in vivo receptor occupancy in the hypothalamus at 4 h after a single oral administration of 3, 30, and 300 mg/kg were 31.7±11.3%, 74.7±11.3%, and 79.8±4.8%, respective (Supplemental Figure 1C) demonstrating sunobinop occupies brain NOP receptors in vivo following oral dosing.

Sunobinop displays a favorable pharmacokinetic profile in rats

After oral administration at 0.3, 1, 3, 10 and 30 mg/kg, the plasma concentration of sunobinop reached a maximum at 2.50–4.50 h with corresponding means±SE C_{max} values of 17.1±4.5, 39.9±1.3, 154±19, 481±101 and 1370±300 ng/mL and corresponding AUC_{inf} values of 142±45, 371±26, 1500±210, 5010±1030 and 12200±2000 ng·h/mL, respectively (Figure 1A). Both the C_{max} and AUC_{inf} values increased dose-proportionally between 0.3 and 30 mg/kg (Figure 1G and 1H). Sunobinop therefore displays linear pharmacokinetics up to 30 mg/kg. The bioavailability of sunobinop ranged from 31.2 to 42.1% at oral doses of 0.3–30 mg/kg. As such sunobinop displayed favorable pharmacokinetics suitable for subsequent in vivo evaluation following oral administration.

Activation of NOP by sunobinop reduces wakefulness and increases non-REM sleep in rats

We examined the dose response effect of sunobinop on sleep-wakefulness cycles during the nocturnal period (administration at 5PM, 3 h prior to lights off) in male Sprague Dawley rats. Sunobinop at a dose of 3 mg/kg caused a tendency towards decrease in wakefulness and increase in non-REM sleep in first 3 h after administration. Also, a decrease in wakefulness and increase in REM sleep were observed at some of the time points (time course data not shown), but these effects were transient, and no significant changes (p values>0.05) were found in the total amount of each stage at any time period (Figure 1B). Administration of 30 mg/kg of sunobinop induced obvious EEG changes. Specifically, wakefulness was decreased during 3-6 h after administration and the total amount of wakefulness was significantly decreased during the nocturnal ($p<0.01$) and whole recording periods ($p<0.01$). Conversely, non-REM sleep was increased during 2-8 h after administration and the total amount of non-REM sleep was significantly increased across all time periods ($p<0.01$). REM sleep was also decreased through the overall recording periods ($p<0.05$) and the total amount of REM sleep was significantly decreased at all time periods (0-3 h, $p<0.01$; 3-15 h $p<0.05$)) (Figure 1B). Following administration of 300 mg/kg of sunobinop the EEG levels show a decrease in wakefulness from 3 to 8 h after administration, with the total amount

of wakefulness significantly decreased in the nocturnal ($p<0.01$) and whole time periods ($p<0.01$). The percentage of non-REM sleep was increased until 8 h after administration and the total amount of non-REM sleep percentage was significantly increased at all time periods ($p<0.01$). In contrast, percentage of REM sleep was only decreased during 0-3 h ($p<0.01$) and was not decreased during the nocturnal period ($p>0.05$) (Figure 1B).

To confirm that these effects were mediated via activation of NOP, 300 mg/kg of sunobinop was administered to wild type and NOP knockout rats on a Wistar Hannover background. Following administration to wild type Wistar Hannover rats the EEG recordings showed a decrease in percentage of wakefulness (0-3 h, $p<0.05$; 3-9 h $p<0.01$) and an increase in non-REM sleep percentage up to 9 h (0-3 h, $p<0.01$; 3-9 h $p<0.01$; 9-15 h $p<0.05$) (Supplemental Figure 2B) in line with results from Sprague Dawley rats (Figure 1B). REM sleep percentage was also decreased only during the first 3 h post-dose (prior to lights out) ($p<0.01$). In contrast, when sunobinop was administered to NOP knockout rats, the EEG changes observed in wild type rats were nearly abolished (Figure 1B and Supplemental Figure 2B). A slight increase in percentage of wakefulness was observed at 3 disparate points for wakefulness and one isolated point for non-REM during the recording period ($p<0.05$) There was no overall statistically significant effect ($p>0.05$) for wakefulness, non-REM or REM sleep % at 0-3, 3-9 and 9-15 h post-dose (Supplemental Figure 2B). Zolpidem (10 mg/kg, p.o.), a nonbenzodiazepine GABA(A) receptor agonist sedative hypnotic, induced a short-lived decrease in wakefulness (0-3 h, $p<0.05$) and an increase in non-REM sleep (0-3 h, $p<0.01$) only during the first 3 h after dosing in NOP knockout rats, indicating an intact and functional of GABAergic system in the knockouts (Supplemental Figure 2C). These results demonstrate that sunobinop produces dose-dependent and statistically significant decrease in wakefulness and increase in non-REM sleep via activation of NOP.

Activation of NOP by sunobinop does not affect respiratory parameters or gastrointestinal motility

No treatment-related changes were noted in respiratory rate at any dose level of sunobinop or at any timepoint post-dosing ($p>0.05$) (Supplemental Figure 1D). As such, sunobinop does not affect the respiratory system in rats following oral doses of up to 600 mg/kg.

In the charcoal meal assay of small intestinal transit, no significant differences were found in the relative intestinal transit between sunobinop and vehicle treated groups ($p>0.05$). The relative intestinal transit rates were 53.5, 57.5, 56.8 and 53.6% in the sunobinop 10, 60, 200 and 600 mg/kg groups, respectively (Figure 3C). In contrast, morphine produced a significant inhibition as compared to vehicle ($p<0.05$), the relative intestinal transit being 13.1% (Supplemental Figure 1E). As such sunobinop did not affect small intestinal transit in rats at doses up to 600 mg/kg.

Activation of NOP by sunobinop does not affect learning/memory and does not produce rewarding effects in rats

The Morris water maze was used to determine if activation of NOP receptors produced deficits in spatial learning and memory. Sunobinop did not produce statistically significant deficits on escape latency as compared to vehicle at any time point ($p<0.05$) (Figure 1C). In contrast, both triazolam at 1 mg/kg and zolpidem at 10 mg/kg produced statistically significant deficits in escape latency on days 3 and 5 and 3, 4 and 5 ($p<0.05$), respectively (Figure 1C) with zolpidem producing a greater deficit than triazolam (escape latency for zolpidem 77, 70, 64 and 64 seconds on days 2, 3, 4 and 5 respectively; escape latency for triazolam 72, 55, 39 and 32 seconds on days 2, 3, 4 and 5 respectively). As such sunobinop does not affect spatial learning and memory as measured by the Morris water maze at doses up to 600 mg/kg while triazolam at 1 mg/kg and zolpidem at 10 mg/kg impaired spatial learning and memory to some extent. Importantly the doses of sunobinop used are substantially above those required for a therapeutic effect.

The conditioned place preference assay was used to determine if sunobinop produced rewarding effects in rats. Sunobinop did not produce a statistically significant place preference as compared to vehicle up to 300 mg/kg (the highest dose tested) ($p<0.05$). A non-statistically significant decreasing trend was noted (drug-vehicle of 151, 49, 22 seconds for vehicle, 30 mg/kg and 300 mg/kg sunobinop respectively) (Figure 1D). Importantly, an aversive effect (which would be indicated by negative numbers) was not evident. In contrast a rewarding effect was noted following conditioning with 8 mg/kg of oxycodone administered subcutaneously (s.c.) (drug-vehicle of 232 seconds) ($p<0.05$) (Figure 1D). As such these data suggest that sunobinop was neither rewarding nor aversive in the rat at the doses which are substantially above those required for a therapeutic effect.

Sunobinop displays a favorable pharmacokinetic profile and produces somnolence in healthy human volunteers

Safety, tolerability and pharmacokinetics were evaluated in a first-in-human trial with healthy male human subjects following oral administration of sunobinop. Mean sunobinop plasma concentration-time profiles were established following administration of 3, 10 and 30 mg oral doses (Figure 1E). Sunobinop exhibited favorable rapid absorption from an aqueous suspension in methyl cellulose across a wide dose range. Time to maximum plasma concentration (T_{max}) was similar across the 3 doses and ranged from 1 to 3 h. The terminal elimination half-life of sunobinop ranged from 2.1 to 3.2 h suggesting suitability for once-daily dosing at nighttime with low concentrations the day after dosing. Beyond 10 mg, systemic exposure increased less than dose proportionally (Figure 1E) with a lower percentage of unchanged drug recovered in urine (70% at 10mg and 28% at 30mg as compared to 89% at 3mg) and no detectable levels of metabolites identified in plasma or urine, suggesting dose-limiting absorption and a predominantly renal route of excretion of absorbed drug.

Sunobinop was generally well tolerated across the range of doses (3 - 30 mg) in healthy male subjects (Supplemental Table 1A). There were no deaths, serious adverse events (SAEs), or discontinuations due to adverse events (AEs) in the study. The most commonly reported treatment emergent events were fatigue/somnolence, euphoria, and dizziness. Fatigue and/or somnolence lasting 3.5-27 h was observed in 3 of 4 subjects at 3 mg, all 8 subjects in the combined 10 and 30 mg dose cohorts vs. in none of the 6 subjects dosed with placebo. Following morning dosing, some subjects receiving sunobinop 10 mg (1 of 4 subjects) and 30 mg (1 of 4 subjects) experienced somnolence sufficient to interfere with activities of daily living. The AE profiles suggested marked CNS effects that increased in incidence, duration, and intensity with increasing systemic exposure.

Activation of NOP by sunobinop increases sleep efficiency and non-REM sleep while decreasing latency to persistent sleep and wake time after sleep onset in patients with insomnia disorder

The impact of sunobinop on sleep wake function was established using polysomnography in a sleep-lab study of patients with insomnia disorder. Sleep Efficiency (SE), the primary endpoint as measured by 8 h of continuous polysomnography monitoring, was defined as the ratio of total sleep time (TST) to time in bed (TIB, 8 h) multiplied by 100 to yield a percentage. Sleep efficiency after dosing with sunobinop was significantly higher than after placebo (91.4% versus 79.8%, respectively). The drug-effect difference between sunobinop and placebo was 11.8% ($p<0.0001$) (Figure 1F). Sunobinop also produced a reduction in the time to fall asleep (latency to persistent sleep (LPS)) ($p=0.0136$), less time awake after sleep onset (wake after sleep onset, WASO) ($p<0.0003$) and fewer nighttime awakenings ($p<0.0001$) (Figure 1F). Sleep stage analysis revealed little-to-no change in the placebo-treated subjects while sunobinop-treated subjects had less stage N1 sleep, more stage N2 sleep, a reduced REM period and no significant change on stage N3 sleep or REM latency (Figure 1G). Sunobinop also increase perceived sleep quality ($p=0.002$) (Figure 1H).

No subjects experienced serious treatment-emergent AEs (TEAEs). Overall, 16 (72.7%) subjects reported at least 1 TEAE, all of which were in the Nervous System Disorders system organ class. Most reported TEAEs occurred during the sunobinop treatment period compared with the placebo treatment period (15 [78.9%] subjects versus [9.5%] subjects, respectively). Treatment-emergent AEs reported during the sunobinop treatment period were somnolence (13 [68.4%] subjects), sedation (2 [10.5%] subjects), and headache (1 [5.3%] subject) (Supplemental Table 1B). Two TEAEs were reported during the placebo treatment period, occurring in 1 (4.8%) subject each: headache and presyncope. All but 2 TEAEs were mild in intensity, and none were assessed as severe. During the placebo treatment period, 1 (4.8%) subject had an AE of presyncope that led to study drug withdrawal and discontinuation from the study. There were no AEs leading to study drug withdrawal or discontinuation from the study after treatment with sunobinop. No clinically significant changes from baseline of treatment period 1 or treatment period 2 were observed during the placebo or sunobinop treatment periods in hematology, chemistry, and urinalysis results (with no evidence of drug-related crystalluria or hematuria). During the sunobinop treatment period, 4 (21.1%) subjects and 3 (15.8%) subjects, respectively, had clinically notable systolic (<90mmHg) and diastolic (≤50mmHg) postbaseline (post-lights-out) blood pressure values, compared with 1 (5.3%) subject with a clinically notable systolic blood pressure value at baseline. Importantly no meaningful changes from baseline were observed in ECGs and SpO_2 measurements during the study. There were no clinically significant ECG findings. No subjects had suicidal ideation or behavior at baseline or during the study, as determined through administration of the Columbia-Suicide Severity Rating Scale.

Author contributions: Conceptualization: GTW, DJK, MU, S Harris, GZ, SW. Methodology: GTW, RK, MZ, AC, MS, MH, TK, KF, YM, S Hiroyama, NT, GZ, SW. Investigation: RK, MZ, AC, EH, MS, MH, TK, KF, YM, S

Hiroyama, NT, GZ, SW. Analysis and Visualization: MS, MH, TK, KF, YM, S Hiroyama, NT, GZ, SW. Supervision: GTW, DJK, MU, S Harris, GZ, SW. Writing: GTW, DJK, RK.

Acknowledgments: All funding was provided by Imbrium Therapeutics/Purdue Pharma L.P. and Shionogi & Co. We thank V. Pascale for helpful editing of the manuscript. We also thank all the recruited human participants for participating in this study.

Supplemental References

1. Zhang C, et al. Novel, potent ORL-1 receptor agonist peptides containing alpha-Helix-promoting conformational constraints. *J Med Chem.* 2002;45(24):5280-6.
2. Berthele A, et al. [3H]-nociceptin ligand-binding and nociceptin opioid receptor mrna expression in the human brain. *Neuroscience.* 2003;121(3):629-40.
3. Quigley DI, et al. Integrity of tritiated orphanin FQ/nociceptin: implications for establishing a reliable binding assay. *Peptides.* 2000;21(7):1111-8.
4. Pike VW, et al. Synthesis and evaluation of radioligands for imaging brain nociceptin/orphanin FQ peptide (NOP) receptors with positron emission tomography. *J Med Chem.* 2011;54(8):2687-700.
5. Kales AaR, A. *A manual of standardized terminology, techniques and scoring system for sleep stages of human subjects.* University of California, Los Angeles: National Institute of Neurological Diseases and Blindness (U.S.)1968.
6. Roth T, et al. A 2-night, 3-period, crossover study of ramelteon's efficacy and safety in older adults with chronic insomnia. *Current medical research and opinion.* 2007;23(5):1005-14.

Supplemental Figure 1

A

	K_i nM (SEM)	GTP EC50 nM (SEM)	GTP E_{max} % (SEM)	Description
**NOP**	**3.3 (0.4)**	**4.03 (0.86)**	**47.8 (1.31)**	**High affinity, high potency partial agonist**
**mu**	1630 (76.5)	>20000	–	Low affinity antagonist
**kappa**	2280 (213)	>20000	–	Low affinity antagonist
**delta**	4763 (509)	2205 (396.6)	16.4 (0.75)	Low affinity, low potency partial agonist

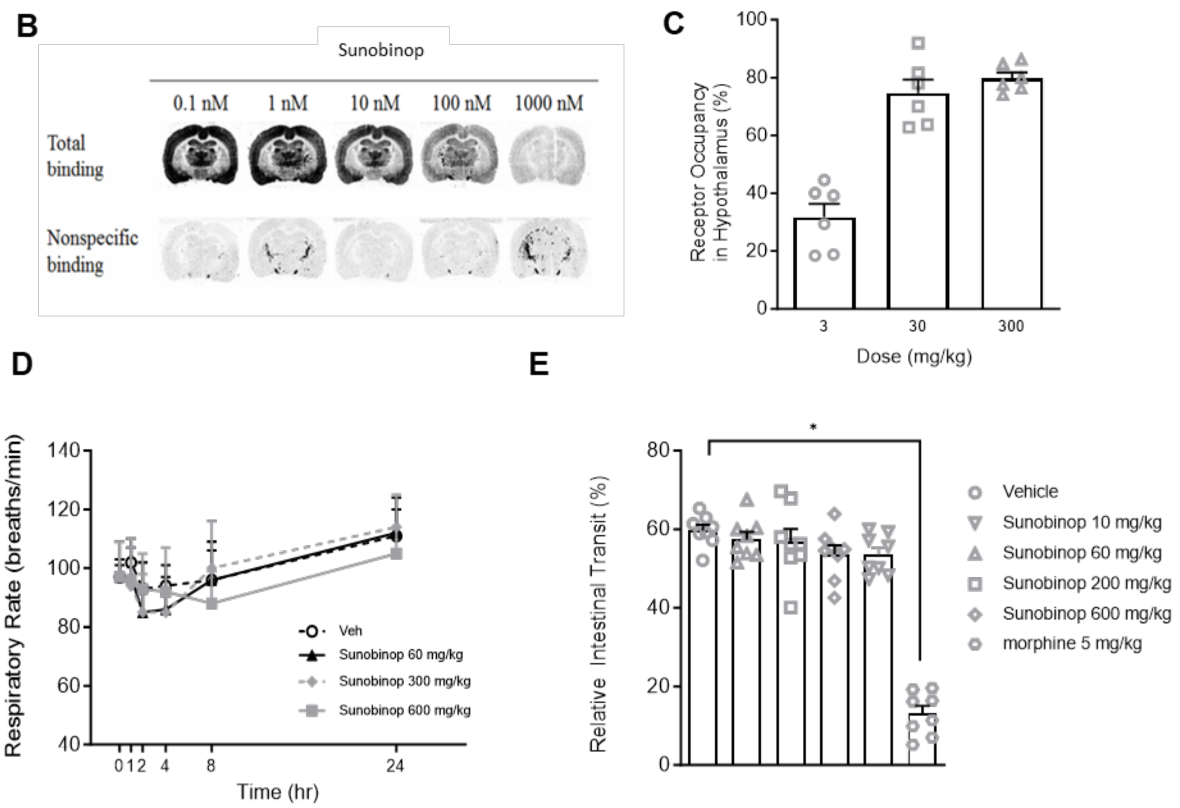

Supplemental Figure 1. Sunobinop pharmacological characterization in vitro, ex vivo and in vivo in rats. Sunobinop is a high affinity partial agonist at human NOP receptors without agonist activity at mu or kappa receptors and with weak partial agonist activity at delta receptors (A). Sunobinop (0.1-1000 nM) competitively inhibited [^{3}H]-NOP-1A binding on rat coronal brain sections with an IC_{50} of 7.7 nM (B) and achieved high receptor occupancy following oral dosing (3-300 mg/kg) to rats (C). Sunobinop, 60, 300 and

600 mg/kg was administered p.o. and no treatment-related changes were noted in the respiratory rate (D) or on intestinal transit of a charcoal meal (E). All data mean±SEM; * $p \leq 0.05$ (Dunnett's test, D; t-test, E).

Supplemental Figure 2

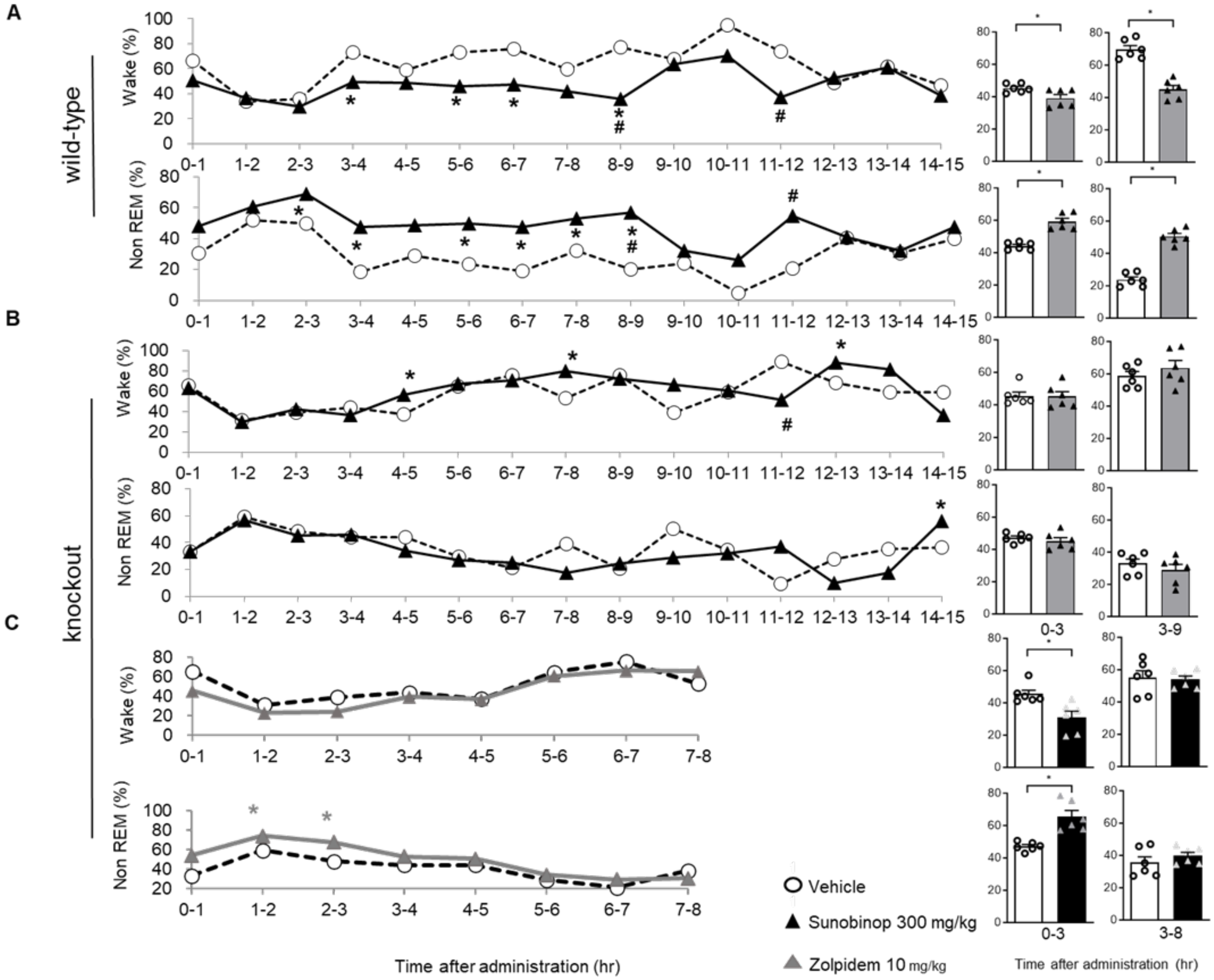

Supplemental Figure 2. Activation of NOP decreases wakefulness and increase non-REM sleep in rats.

Sunobinop (300 mg/kg) was administered p.o. during the nocturnal period and produced dose-dependent and statistically significant decrease in wakefulness and increase in non-REM sleep (A). The duration of effect on wakefulness and non-REM sleep was 6-7 h between 2 and 9 h post-administration (A). Cumulative effects on wakefulness and non-REM as measured between 0-3 h (when lights were on), and 3-9 h (the nocturnal period) post administration was statistically significantly different from vehicle (A scatter plots). In contrast when sunobinop was administered to NOP knock-out rats (B) there was no

statistically significant difference between sunobinop and vehicle treated rats in the cumulative % of time spent awake or in non-REM indicating these effects are via NOP (B scatter plots). Zolpidem (10 mg/kg) induced a short-lived decrease in wakefulness and an increase in non-REM sleep only during the first 3 h after dosing in NOP knockout rats, indicating an intact and functional of GABAergic system in the knockouts (C). All data mean±SEM, *$p<0.05$ unadjusted t-test; # $p<0.05$ t-test with Bonferroni correction.

Supplemental Table 1

A

Summary of Distinct Subjects in the Randomized Safety Population With Treatment-Emergent Adverse Events by System Organ Class and Preferred Term

System Organ Class<br>Preferred Term	Sunobinop Dose Level									
	Placebo (N = 6)		3 mg (N = 4)		10 mg (N = 4)		30 mg (N = 4)		Overall (N = 18)	
	n	(%)	n	(%)	n	(%)	n	(%)	n	(%)
Any TEAE	1	(16.7)	4	(100.0)	4	(100.0)	4	(100.0)	13	(72.2)
Gastrointestinal Disorders	0	(0.0)	1	(25.0)	0	(0.0)	0	(0.0)	1	(5.6)
Constipation	0	(0.0)	1	(25.0)	0	(0.0)	0	(0.0)	1	(5.6)
General Disorders and Administration Site Conditions	0	(0.0)	3	(75.0)	4	(100.0)	2	(50.0)	9	(50.0)
Fatigue	0	(0.0)	3	(75.0)	4	(100.0)	2	(50.0)	9	(50.0)
Nervous System Disorders	1	(16.7)	3	(75.0)	1	(25.0)	4	(100.0)	9	(50.0)
Disturbance in Attention	0	(0.0)	0	(0.0)	0	(0.0)	1	(25.0)	1	(5.6)
Dizziness	0	(0.0)	2	(50.0)	1	(25.0)	2	(50.0)	5	(27.8)
Headache	1	(16.7)	2	(50.0)	0	(0.0)	1	(25.0)	4	(22.2)
Somnolence	0	(0.0)	0	(0.0)	0	(0.0)	3	(75.0)	3	(16.7)
Psychiatric Disorders	0	(0.0)	1	(25.0)	1	(25.0)	2	(50.0)	4	(22.2)
Euphoric Mood	0	(0.0)	1	(25.0)	0	(0.0)	2	(50.0)	3	(16.7)
Nightmare	0	(0.0)	0	(0.0)	1	(25.0)	0	(0.0)	1	(5.6)
Vascular Disorders	0	(0.0)	0	(0.0)	1	(25.0)	0	(0.0)	1	(5.6)
Hypotension	0	(0.0)	0	(0.0)	1	(25.0)	0	(0.0)	1	(5.6)

B

Summary of Distinct Subjects in the Randomized Safety Population With Treatment-Emergent Adverse Events by System Organ Class and Preferred Term

System Organ Class<br>Preferred Term	Placebo (N = 21) n (%)	Sunobinop (N = 19) n (%)	Overall (N = 22) n (%)
Any TEAE	2 (9.5)	15 (78.9)	16 (72.7)
Nervous system disorders			
Somnolence	0	13 (68.4)	13 (68.4)
Headache	1 (4.8)	1 (5.3)	2 (9.1)
Sedation	0	2 (10.5)	2 (9.1)
Presyncope	1 (4.8)	0	1 (4.5)

Supplemental Table 1. Summary of safety information in healthy human volunteers and in patients with Insomnia disorder.

Sunobinop was generally well tolerated and safe across the range of doses (3 – 30 mg) in healthy male subjects (A) and in patients with insomnia disorder (B) with the most prominent CNS effects being somnolence/fatigue.
